# Prognostic Value of Blood Urea Nitrogen to Albumin Ratio in Elderly Critically Ill Patients with Acute Kidney Injury: A Retrospective Study

**DOI:** 10.3390/medicina61071233

**Published:** 2025-07-08

**Authors:** Sinem Bayrakçı, Elif Eygi

**Affiliations:** 1 Department of Intensive Care Unit, Gaziantep City Hospital, 27470 Gaziantep, Türkiye; 2 Department of Anesthesiology and Reanimation, GaziantepCity Hospital, 27470 Gaziantep, Türkiye; drelifeygi6@gmail.com

**Keywords:** elderly, acute kidney injury, intensive care unit, mortality, BUN/albumin ratio

## Abstract

*Background and Objectives*: Acute kidney injury (AKI) is common in intensive-care unit (ICU) patients and is associated with increased mortality. Elderly patients tend to have more comorbid chronic diseases and are more prone to AKI than younger populations, resulting in higher rates of hospitalization and a higher incidence of AKI. Our aim in this study was to investigate the prognostic utility of BUN/albumin ratio (BAR) in predicting mortality in elderly critically ill patients with AKI. *Materials and Methods*: This study was conducted retrospectively on 154 elderly patients with AKI who were admitted to the ICU between October 2023 and September 2024.Data on the following demographic, clinical, and laboratory parameters were retrospectively collected from medical cards and electronic records. Results: In the non-survivor group, among comorbidities, lung disease was higher (*p* < 0.05), GCS was lower, and APACHE II was higher among clinical scores (*p* < 0.001). In the non-survivor group, diuretic use (*p* = 0.03), oliguria, RRT, vasopressor requirement, sepsis, and MV rates (*p* < 0.001),as well as BUN, phosphate, LDH, Crp, APTT, INR, and BAR rates, were higher (all *p* < 0.05) and albumin was lower (*p* = 0.01). Cut-off values of BUN, albumin, and BAR variables according to mortality status were determined by an ROC curve analysis, as follows:48.4 for BUN (*p* = 0.013), 31.5 for albumin (*p* = 0.001), and 1.507 for BAR (*p* = 0.001).According to the results of the ROC analysis performed to predict in-hospital mortality, the BAR level reached an AUC value of 0.655. A BAR value above 1.507 increases mortality by 3.944 times (*p* = 0.023). *Conclusions*: BAR is a simple and accessible biomarker that may serve as a predictor of in-hospital mortality in elderly patients with AKI. Its use may aid early risk stratification and decisionmaking in the ICU.

## 1. Introduction

Acute kidney injury (AKI) is a common kidney disease that causes deterioration in kidney function and a sudden decrease in glomerular filtration [1]. AKI is common in intensive-care unit (ICU) patients and is associated with increased mortality. AKI is also associated with prolonged hospital stays and increased healthcare costs [2].

With the increase in the number of elderly individuals in the global population, the ages of patients admitted to the ICU have increased in recent years. Elderly patients tend to have more comorbid chronic diseases and are more prone to AKI than younger populations, resulting in higher rates of hospitalization and a higher incidence of AKI. The prognosis of AKI in elderly patients is poor, and the mortality rate is higher in elderly patients with AKI in the ICU [3,4].

Given the high incidence of AKI and its poor outcome in critical illness, it is particularly important to identify predictors to predict prognoses, and, thus, many observational studies have been conducted to search for reliable predictors of mortality in AKI [5,6].

Blood urea nitrogen (BUN) is the primary metabolite of human protein. BUN is a biomarker used to assess hypovolemia and renal function. BUN is a crucial metric that shows how renal health, protein metabolism, and nutritional status are related. It rises when the glomerular filtration rate falls [7]. An increased BUN level has been found to be a predictor of mortality in elderly people [8].

Albumin originates from the liver and is the main protein of the blood. Albumin reflects the nutritional status of the body and has various physiological properties, such as antioxidant and anti-inflammatory [9]. In elderly patients, a lower albumin level was linked to both readmission to the hospital and all-cause mortality [10].

The BUN/albumin ratio (BAR), which combines two routine laboratory values, combines the nutritional status of organs and renal status. Studies on BAR, a current biomarker, have shown that BAR is a prognostic predictor of AKI and in-hospital mortality in patients with intracranial hemorrhage (ICH) [11], is associated with mortality in patients with AKI [12], is a predictor of ICU admission and mortality in geriatric patients with gastrointestinal bleeding [13] and is also a predictor of the need for renal replacement therapy in patients with acute renal failure due to COVID-19 pneumonia [14].

Although many studies have been conducted to estimate the prognosis and predict mortality in critically ill patients with AKI, the number of studies conducted in critically ill elderly patients, who are more prone to AKI andhave a worse prognosis and a higher mortality rate than younger populations, is limited. We believe that a low-cost and easily calculated biomarker that can predict prognosis in critically ill elderly patients with AKI can also predict mortality in this patient group. Our aim in this study was to investigate the prognostic utility of BAR in predicting mortality in elderly critically ill patients with AKI.

## 2. Materials and Methods

### 2.1. Study Design and Patient Population

This retrospective observational study was conducted between October 2023 and September 2024 in three anesthesia and reanimation ICUs of Gaziantep City Hospital. The Institutional Research Ethics Committee approved the study, protocol number: 61/2024.

**Figure 1:**
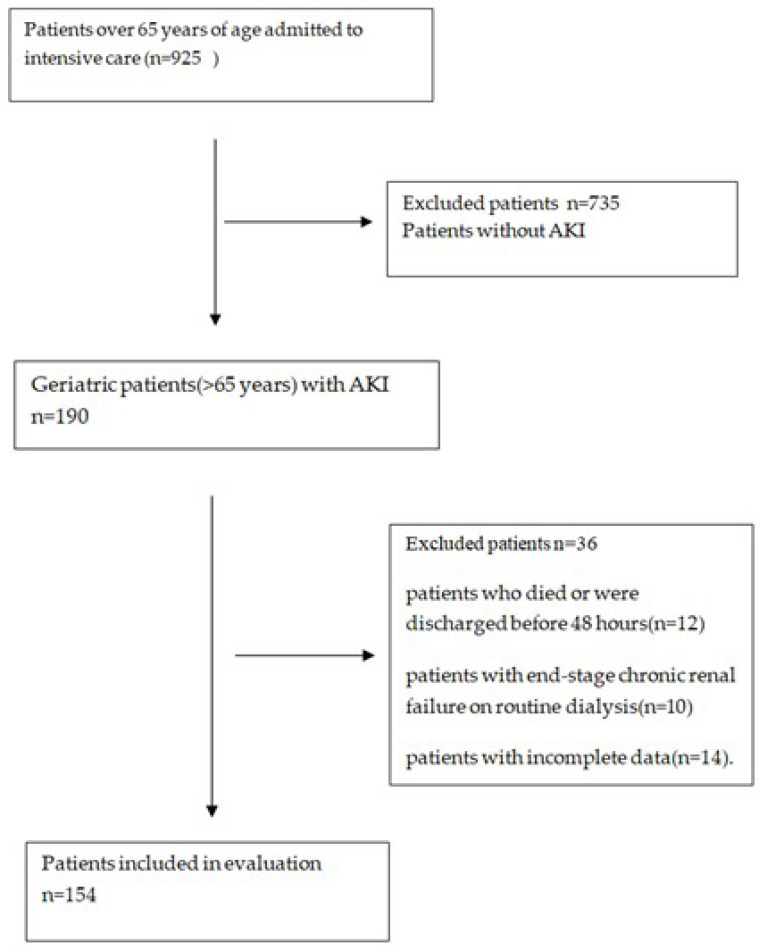
Flow chart of the study.

All elderly patients (>65 years old) who were followed up in the ICU and developed AKI on admission were included in the study (n = 190). The occurrence of AKI was determined on the basis of the Kidney Disease: Improving Global Outcomes (KDIGO) definition. For inclusion, patients needed to be hospitalized in the ICU at first admission for more than two days. The exclusion criteria were as follows: patients who died or were discharged before 48 h (n = 12), patients with end-stage chronic renal failure on routine dialysis (n = 10), and patients with incomplete data (n = 14). At last, 154 patients were enrolled in the study. Figure 1 shows aflowchart of the study.

The patients who died were defined as non-survivors and the others as survivors, and these two groups were compared. The primary outcomes of the study were to predict in-hospital mortality in elderly critically ill patients with AKI using BUN, albumin, and BAR values and to investigate whether BAR has high predictive power for mortality. A receiver operating characteristic (ROC) analysis was performed to determine the in-hospital mortality predictive power of the BUN, albumin, and BAR levels. Based on the optimal cut-off value, we divided the whole cohort of patients into two groups.

### 2.2. Data Collection

Data on the following demographic, clinical, and laboratory parameters were retrospectively collected from medical cards and electronic records: age, gender, comorbid diseases, severity scores as determined by the Acute Physiology and Chronic Health Evaluation (APACHE) II, Glasgow Coma Scale (GCS), oliguria, diuretic use, hospital and intensive-care length of stay (LOS), dialysis/renal replacement therapy (RRT) requirement, sepsis, vasopressor requirement, mechanical ventilation (MV) requirement, and mortality. Laboratory tests included neutrophil percentage, albumin, bicarbonate, lactate, creatinine, glucose, hematocrit, hemoglobin, platelet, sodium, potassium, C-reactive protein (Crp), blood urea nitrogen (BUN), LDH, white blood cell (WBC), activated partial thromboplastin time (APTT), and international normalized ratio (INR). BUN/albumin (BAR) was calculated as the BUN/albumin ratio.

### 2.3. Statistical Analysis

While evaluating the findings obtained in the study, the SPSS (Statistical Package for Social Sciences for Windows) 27.0 program was used for the statistical analysis. Descriptive statistics of the continuous variables areexpressed as the mean and standard deviation, and the descriptive statistics of the categorical data areexpressed as the frequency and percentage. An independent sample t-test and Mann–Whitney U test were used to compare the quantitative data. The chi-square test was used in the relationship analysis of the categorical data. In addition, the ROC curve was used to determine the cut-off, and binary logistic regression analysis was used to estimate the mortality status.

## 3. Results

The study involved 154 patients. The average age was 76.76 ± 7.28 years, of whom 50.0% were male. The mortality rate was 44.1% (n = 68); these patients were defined as non-survivors. Clinical data of the two groups of patient (the survivor group and the non-survivor group) were compared in Table 1. In the non-survivor group, lung disease was higher among comorbidities (*p* < 0.05), while GCS was lower and APACHE 2 was higher among clinical scores (*p* < 0.001). As shown in Table 1, diuretic use (*p* = 0.03), oliguria, RRT, vasopressor requirement, sepsis, and MV rates were higher in the non-survival group (*p* < 0.001). The BUN, phosphate, LDH, Crp, APTT, INR, and BAR ratio were higher in the non-survival group (all *p* < 0.05), while albumin was lower (*p* = 0.01).

**Figure 2:**
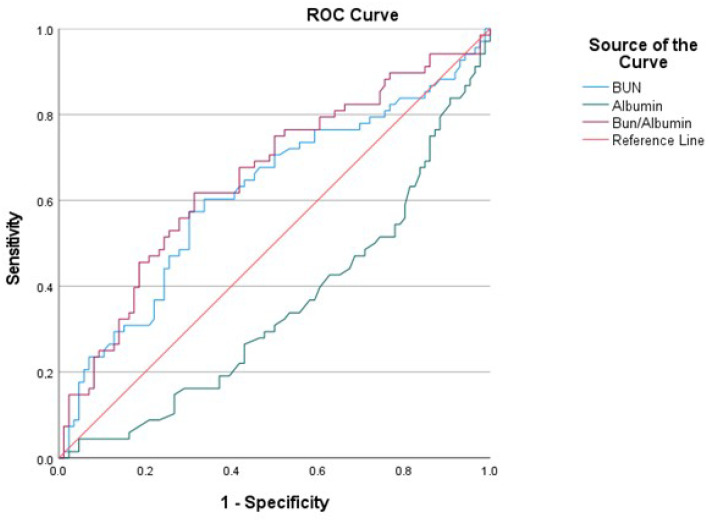
Roc curve results.

According to the ROC curve analysis of BAR, 1/ALB and BUN to predict in-hospital mortality, the area under the curve (AUC) of the BAR ratio for in-hospital mortality was 0.65 (95% CI 0.567–0.743), and the cut-off value was 1.507, with sensitivity of 0.6176 and specificity of 0.6163 (Figure 2). In addition, the sensitivity and specificity values of 1/ALB and BUN for in-hospital mortality are presented in Table 2. We divided the patients into two groups according to their BUN/albumin ratio. There were 75 patients with a BUN/alb ratio of ≥1.507 and 79 patients with a BUN/alb ratio of <1.507.

As seen in Table 3, there was a significant difference between the two groups in terms of APACHE 2 score, RDW-SD, RDW-CV, BUN, creatinine, potassium, magnesium, phosphate, albumin, Crp, APTT, INR, hypertension, oliguria, RRT used, vasopressor requirement, sepsis, and mechanical ventilation treatment.

The results of the correlation analysis of the mortality status according to the determined cutoffs are given in Table 4. While the BUN value of 40.7% (n = 35) of the survivors was 48.4 and above, 60.29% (n = 41) of the deceased were distributed as 48.4 and above (*p* = 0.016). The albumin value of 60.47% (n = 52) of the survivors was 31.5 and above, while 39.71% (n = 27) of the deceased were distributed as 31.5 and above (*p* = 0.010). The BUN/albumin value of 38.37% (n = 33) of the survivors was 1.507 and above, while 61.76% (n = 42) of the deceased were distributed as 1.507 and above (*p* = 0.004).

Variables that have a significant relationship with mortality status were affected separately (univariate) and binary logistic regression analysis was performed. As a result of the analysis, those with BUN values of 48.4 and above are 2.213 times more likely to die than those below (*p* = 0.016). Those with albumin values of 31.5 and below are 2.322 times more likely to die than those above 31.5 (*p* = 0.011). Those with BUN/albumin values of 1.507 and above are 2.594 times more likely to die than those below (*p* = 0.004). Those without lung disease are 2.538 times more likely to die than those with (*p* = 0.031). Those with oliguria (first day) are 3.969 times more likely to die than those without (*p* < 0.001). Those with diuretic use are 2.034 times more likely to die than those without (*p* = 0.031). Those with RRT used are 6.282 times more likely to die than those without (*p* = 0.001). Those with vasopressor requirements are 143.364 times more likely to die than those without (*p* < 0.001). Those with sepsis are 5.458 times more likely to die than those without (*p* < 0.001). Those with mechanical ventilation are 75.614 times more likely to die than those without (*p* < 0.001).

A general model was established by simultaneously affecting all significant variables (Table 5). The variables that were insignificant as a result of the model (BUN, albumin, lung disease, oliguria (first day), diuretic use, RRT used, and sepsis) were removed, and the general model was established with the significant variables BAR, vasopressor requirement, and mechanical ventilation.

As a result of the established model, those with a BAR value above 1.507 had an increased risk of death by 3.944 times (*p* = 0.023), those with a vasopressor requirement by 21.067 times (*p* = 0.001), and those using mechanical ventilation by 37.672 times (*p* < 0.001).

## 4. Discussion

This study demonstrates that the BUN/albumin ratio is a significant predictor of in-hospital mortality in elderly ICU patients with AKI. To our knowledge, this is the first study specifically exploring the prognostic value of BAR in elderly critically ill patients with AKI.

AKI has a high mortality and morbidity rate. With age, structural and functional changes in the kidney make elderly patients more prone to kidney failure. Therefore, AKI is common in elderly patients and is an important health problem with a high mortality rate. Various studies have shown that the mortality rate of AKI in elderly patients varies between 20% and 61% [15,16,17]. In our study, the mortality rate in elderly patients with AKI was 44.16%. One of the reasons for this change in mortality rates in studies in the literature is the variability in the age groups of patients included in the study. In our study, we included the elderly patient group as those over 65 years of age. Another reason for the variability in mortality rates is due to the exclusion of intensive care patients in some studies.

In their study examining critically ill patients with AKI, Shi et al. reported a higher rate of first-day oliguria in the non-surviving group (*p* < 0.05) [18]. Similarly, in our study, first-day oliguria was higher in the non-survivor group (*p* < 0.001). In astudy by Gong et al. there was no significant difference in diuretic use and RRT use between survivors and non-survivors among elderly patients with AKI (*p* = 0.666, *p* = 0.070) [19]. In contrast, in our study, diuretic and RRT use were higher in the non-survivors group (*p* = 0.030, *p* < 0.001). Gong et al. found significant differences in mechanical ventilation and dopamine use between the survivor and non-survivor groups of elderly AKI patients [19]. In astudy by Shi et al., the need for noradrenaline, mechanical ventilation, and RRT was significantly higher in the non-survivor group (*p* < 0.001) [18]. Supporting their study, in our study, the need for mechanical ventilation and vasopressors was higher in the non-survivor group (*p* < 0.001).

Contrary to studies in the literature, in our study, no significant difference was found between the surviving and non-surviving groups in terms of length of hospital stay and intensive-care unit stay [19,20].

BUN is the nitrogen component of urea, the end product of protein metabolism, originating in the liver and excreted by the kidneys [7]. It is released in large amounts when renal perfusion is inadequate and renal function is impaired, which may better reflect the severity of renal damage. BUN is an important indicator of dehydration status and is often used as a biomarker for renal function and hypovolemia. BUN may also trigger immune dysfunction by activation of neurohumoral mechanisms, thereby increasing the risk of death in critically ill patients with AKI [21]. Studies have shown that BUN levels are a risk factor for the need for renal replacement therapy in AKI patients, and increases in BUN are prognostically associated with mortality in AKI patients [22]. In our study, BUN levels were significantly higher in the non-surviving group than in the surviving group. In addition, according to the results of ROC analysis performed to predict in-hospital mortality, BUN levels reached an AUC value of 0.617. Those with BUN values of 48.4 mg/dL and above were 2.213 times more likely to die than those with values below (*p* = 0.016).

Albumin is a protein synthesized in the liver. Albumin is a nutritional status marker and also has antioxidant and anti-inflammatory effects [23,24]. Hypoalbuminemia is common in critically ill patients and has been associated with increased mortality in studies [25,26]. A meta-analysis reported that hypoalbuminemia is a dose-dependent predictor of adverse outcomes such as mortality, morbidity, and prolonged intensive care and hospital stay [26]. Hypoalbuminemia leads to the body’s inability to effectively remove toxic substances, decreased vascular volume, and renal hypoperfusion, all of which lead to renal injury [27]. Murashima et al. found that hypoalbuminemia was independently associated with the development of postoperative AKI and a high mortality rate in this patient group [28]. A meta-analysis reported that low serum albumin levels were independent predictors of AKI and mortality [29]. In their meta-analysis evaluating the relationship between serum albumin level and the development of AKI and the effect of lower serum albumin on mortality in patients with AKI, Wiedermann et al. provide evidence that hypoalbuminemia is a significant independent predictor of both AKI and death after AKI [29]. In our study, albumin levels were significantly lower in the non-surviving group than in the surviving group. In addition, according to the results of the ROC analysis performed to predict in-hospital mortality, albumin levels reached an AUC value of 0.651. Those with albumin values below 31.5 were 2.322 times more likely to die than those with albumin values of 31.5 and above (*p* = 0.011).

In addition to being a sign of decreased renal clearance, elevated BUN levels can also be a sign of catabolic stress, gastrointestinal bleeding, hypovolemia, and accelerated protein turnover—all of which are linked to worse outcomes for patients in critical condition. Conversely, serum albumin is a negative acute phase reactant that falls in response to malnourishment and inflammation, both of which are prevalent in older intensive-care unit patients. The BUN/Alb ratio may combine these two parameters to provide a more holistic assessment, serving as a composite marker of both renal function and systemic disease severity. Therefore, BAR may be a combined indicator of systemic physiological stress and organ dysfunction. This ratio may have a more pronounced effect on mortality, especially in elderly patients, considering the decreased physiological reserve and the frequency of comorbid conditions.

Studies have been conducted investigating BAR, obtained by the ratio of BUN and albumin, as a composite biomarker for various conditions. In the literature, BAR has recently emerged as a potential biomarker for predicting mortality, especially in sepsis, pneumonia, and general intensive care patients. Cheng et al. reported that high BAR values were associated with 28-day mortality in patients with sepsis [30]. Similarly, a study by Dundar et al. reported that high BAR values were associated with in-hospital mortality in older emergency department patients [31]. Pan et al. [32] showed that high BAR levels on admission predicted contrast-induced nephropathy in patients undergoing coronary procedures, while in another study, high BAR levels were associated with in-hospital mortality in cardiac surgery patients [33]. Studies have shown that a high BAR level is associated with mortality in patients with acute respiratory failure and critically ill patients with chronic obstructive pulmonary disease and is associated with both ICU admission and mortality in elderly patients with gastrointestinal bleeding [13,34,35].

He et al. showed that a high BAR is a good diagnostic predictor of AKI in patients with rib fractures in the ICU, while Acehan showed that BAR is a good biomarker for predicting mortality and disease severity as well as the need for RRT in patients with COVID-19 pneumonia who developed AKI [14,36].Yang et al., in their study investigating the prognostic effect of BAR on acute kidney injury and mortality in intensive care patients with intracranial hemorrhage, stated that BAR is a prognostic predictor of AKI and in-hospital mortality in the intensive care unit in patients with ICH [11]. Shi et al., in their study examining the relationship between BAR level and mortality in intensive care patients with AKI, found that BAR is significantly associated with increased all-cause mortality in patients with AKI [12].

In our study, BAR levels were significantly higher in the non-surviving group than in the surviving group. In addition, according to the results of ROC analysis performed to predict in-hospital mortality, the BAR level reached an AUC value of 0.655. A BAR value above 1.507 was associated with a 3.944-fold increased risk of mortality (*p* = 0.023). In our study, we found a relationship between high BAR levels and mortality. Our study contributes to the literature by focusing on the prognostic value of BAR in a specific clinical setting, such as acute kidney injury, in the elderly critically ill patient group.

A major strength of this study is its focus on a homogeneous elderly population with AKI, which allows for more accurate generalization to geriatric ICUs worldwide. In addition, the retrospective design using routinely collected data increases its applicability for application in resource-limited settings.

Despite these strengths, the study has several limitations. First, the retrospective design and single-center nature limit the generalizability of the results. We were unable to control for specific variables such as baseline nutritional status, fluid management strategies, or infection severity. Second, only BUN and albumin admission values were analyzed; dynamic changes in BAR over time may provide additional prognostic insight.

Future research should aim to validate these findings in multicenter, prospective studies and integrate BAR into multimodal prediction models. It would also be clinically useful to look into the predictive function of BAR trajectories (i.e., rising or falling trends) and possible responses to interventions (e.g., nutritional support andrenal replacement therapy).

## 5. Conclusions

Our study showsthat high BAR levels at admission were associated with increased mortality in critically ill elderly patients with AKI. In conclusion, low-cost and easily calculated BAR levels can be used as prognostic biomarkers associated with mortality in critically ill elderly patients with AKI. Its inclusion in routine intensive care assessments may improve early risk stratification and guide management decisions.

## Figures and Tables

**Table 1:** Descriptive statistics regarding patient variables and statistical analyses according to mortality status.

Characteristic	All Patients (n = 154)	Survivors (n = 86)	Non-Survivors (n = 68)	*p*-Value
Clinical parameter, (mean ± SD), n (%)				
Age (years)	76.76 ± 7.28	76.66 ± 6.92	76.88 ± 7.77	0.853
Gender (male)	77 (50%)	41 (47.67%)	36 (52.94%)	0.516
Comorbidity, n (%)				
Diabetes mellitus	57 (37.01%)	36 (41.86%)	21 (30.88%)	0.161
Hypertension	76 (49.35%)	44 (51.16%)	32 (47.06%)	0.613
Heart disease	65 (42.21%)	39 (45.35%)	26 (38.24%)	0.375
Lung disease	33 (21.43%)	24 (27.91%)	9 (13.24%)	0.028
Liver disease	3 (1.95%)	1 (1.16%)	2 (2.94%)	0.428
Neurological diseases	34 (22.08%)	16 (18.6%)	18 (26.47%)	0.243
Malignancy	14 (9.09%)	7 (8.14%)	7 (10.29%)	0.644
Other	5 (3.25%)	3 (3.49%)	2 (2.94%)	0.849
Clinical scores, (mean ± SD)				
GCS	11.87 ± 3.78	13.83 ± 1.88	9.4 ± 4.13	<0.001
APACHE II	20.79 ± 8.73	17.12 ± 6.51	25.43 ± 9.02	<0.001
Treatments/examination, n (%)				
Oliguria (first day)	69 (44.81%)	26 (30.23%)	43 (63.24%)	<0.001
Diuretic use	71 (46.1%)	33 (38.37%)	38 (55.88%)	0.030
RRT used [n (%)]	24 (15.58%)	5 (5.81%)	19 (27.94%)	<0.001
Vasopressor requirement	60 (38.96%)	3 (3.49%)	57 (83.82%)	<0.001
Sepsis	56 (36.36%)	17 (19.77%)	39 (57.35%)	<0.001
Mechanical ventilation	74 (48.05%)	7 (8.14%)	67 (98.53%)	<0.001
Treatments/examination, (mean ± SD)				
ICU LOS (day)	12.71 ± 14.79	9.44 ± 10.62	16.85 ± 18.04	0.092
Hospital LOS (day)	16.9 ± 16.14	15.34 ± 13.5	18.88 ± 18.88	0.940
Laboratory parameters, (mean ± SD)				
WBC	15.8 ± 8.86	15.72 ± 9.67	15.9 ± 7.79	0.898
Hemoglobin (g/L)	11.92 ± 10.61	10.95 ± 2.48	13.13 ± 15.7	0.497
Platelet	257.51 ± 124.58	261.37 ± 123.25	252.63 ± 127	0.667
RDW-SD	51.73 ± 8.95	51.07 ± 9.11	52.57 ± 8.73	0.233
RDW-CV	16.81 ± 3.01	16.68 ± 2.98	16.97 ± 3.05	0.637
BUN (mg/dL)	57.38 ± 43.5	53.08 ± 48.17	62.82 ± 36.37	0.013
Creatinine (umol/L)	2.25 ± 1.35	2.08 ± 0.93	2.46 ± 1.73	0.154
Glucose (md/dL)	177.86 ± 86.65	174.23 ± 84.92	182.46 ± 89.2	0.560
Sodium (mmol/L)	138.69 ± 7.29	137.86 ± 5.98	139.75 ± 8.6	0.126
Potassium (mmol/L)	4.67 ± 0.91	4.75 ± 0.93	4.58 ± 0.9	0.267
Magnesium (mmol/L)	1.94 ± 0.41	1.89 ± 0.39	2.02 ± 0.42	0.072
Phosphate (mmol/L)	4.09 ± 1.88	3.65 ± 1.2	4.71 ± 2.45	0.010
LDH	452.46 ± 574.04	419.29 ± 670.24	494.41 ± 423.52	0.001
Albumin (g/dL)	31.54 ± 6.11	32.93 ± 5.81	29.77 ± 6.07	0.001
C-Reactive protein (mg/dL)	97.13 ± 81.12	83.75 ± 75.61	114.05 ± 85.17	0.004
APTT	30.39 ± 12.31	29.35 ± 12.56	31.69 ± 11.95	0.025
INR	1.45 ± 0.65	1.39 ± 0.55	1.54 ± 0.76	0.007
Lactate (mg/dL)	2.8 ± 2.24	2.43 ± 1.8	3.26 ± 2.63	0.060
Bicarbonate (mmol/L)	21.36 ± 12.23	21.01 ± 4.34	21.81 ± 17.81	0.720
Bun/albumin	1.93 ± 1.45	1.68 ± 1.44	2.25 ± 1.42	0.015

**Table 2:** Roc curve results.

	AUC	SE	%95 CI (Lower–Upper)	Cut-Off	Sensitivity	Specificity	*p*
BUN	0.617	0.047	0.525–0.708	48.4	0.6029	0.5930	0.013
Albumin	0.651	0.044	0.564–0.738	31.5	0.6047	0.6029	0.001
Bun/albumin	0.655	0.045	0.567–0.743	1.507	0.6176	0.6163	0.001

AUC: area under the curve; SE: standard error; cut-off: threshold value.

**Table 3:** Baseline characteristics stratified by BUN/ALB ratio.

Characteristics	BAR < 1.507 (n = 79)	BAR ≥ 1.507 (n = 75)	*p* Value
Clinical parameters, (mean ± SD), n (%)			
Age (years)	76.32 ± 6.57	77.21 ± 7.97	0.566
Gender (male)	41 (52.56%)	36 (47.37%)	0.872
Comorbidity, n (%)			
Diabetes mellitus	30 (38.46%)	27 (35.53%)	0.357
Hypertension	45 (57.69%)	31 (40.79%)	0.004
Heart disease	29 (37.18%)	36 (47.37%)	0.444
Lung disease	18 (23.08%)	15 (19.74%)	0.978
Liver disease	1 (1.28%)	2 (2.63%)	0.530
Neurological diseases	14 (17.95%)	20 (26.32%)	0.181
Malignancy	6 (7.69%)	8 (10.53%)	0.221
Other	3 (3.85%)	2 (2.63%)	0.692
Clinical scores, (mean ± SD)			
GCS	12.05 ± 3.96	11.68 ± 3.6	0.157
APACHE 2	18.49 ± 7.9	23.14 ± 8.97	<0.001
Treatments/examination, n (%)			
Oliguria (first day)	29 (37.18%)	40 (52.63%)	0.006
Diuretic use	35 (44.87%)	36 (47.37%)	0.646
RRT used	8 (10.26%)	16 (21.05%)	0.005
Vasopressor requirement	24 (30.77%)	36 (47.37%)	0.010
Sepsis	26 (33.33%)	30 (39.47%)	0.024
Mechanical ventilation	31 (39.74%)	43 (56.58%)	0.025
Treatments/examination, (mean ± SD)			
ICU LOS (day)	12.67 ± 14.94	12.76 ± 14.74	0.533
Hospital LOS (day)	17.38 ± 16.29	16.41 ± 16.07	0.775
Laboratory parameters, (mean ± SD)			
WBC	15.47 ± 8.96	16.14 ± 8.81	0.765
Hemoglobin (g/L)	11.33 ± 2.37	12.52 ± 14.94	0.684
Platelet	259.92 ± 122.68	255.04 ± 127.27	0.714
RDW-SD	50.03 ± 7.37	53.47 ± 10.08	0.005
RDW-CV	16.19 ± 2.37	17.44 ± 3.44	0.002
BUN (mg/dL)	35.17 ± 8.33	80.19 ± 52.42	<0.001
Creatinine (umol/L)	1.72 ± 0.57	2.8 ± 1.67	<0.001
Glucose (md/dL)	180.42 ± 86.78	175.24 ± 87.01	0.161
Sodium (mmol/L)	138.69 ± 6.67	138.7 ± 7.92	0.471
Potassium (mmol/l)	4.46 ± 0.87	4.9 ± 0.91	0.024
Magnesium (mmol/L)	1.86 ± 0.33	2.03 ± 0.46	0.004
Phosphate (mmol/L)	3.72 ± 1.77	4.45 ± 1.94	0.020
LDH	454.62 ± 655.02	450.25 ± 481.37	0.939
Albumin (g/dL)	33.29 ± 5.32	29.73 ± 6.38	<0.001
C-Reactive protein (mg/dL)	81.98 ± 63.83	112.67 ± 93.58	0.001
APTT	28.44 ± 6.78	32.38 ± 15.94	0.039
INR	1.36 ± 0.46	1.56 ± 0.79	0.036
Lactate (mg/dL)	2.83 ± 2.27	2.76 ± 2.22	0.505
Bicarbonate (mmol/L)	21.37 ± 5.23	21.36 ± 16.64	0.723

**Table 4:** Relationship between mortality status and categorical variables.

		MORTALITY		
		Survival	Ex	*p*
		n (%)	n (%)	
BUN	≥48.4	35 (40.7%)	41 (60.29%)	0.016
Albumin	≥31.5	52 (60.47%)	27 (39.71%)	0.010
Bun/albumin	≥1.507	33 (38.37%)	42 (61.76%)	0.004

**Table 5:** Logistic regression analysis according to mortality status.

	Univariate Analysis		Multivariate Analysis		
	RR (95%CI)	*p*	RR (95%CI)	*p*	R^2^
BUN < 48.4 vs. BUN ≥ 48.4	2.213 (1.156–4.234)	0.016	-	-	0.898
Albumin ≥ 31.5 vs. albumin < 31.5	2.322 (1.212–4.450)	0.011	-	-	
Bun/albumin < 1.507 vs. Bun/albumin ≥ 1.507	2.594 (1.349–4.991)	0.004	3.944 (1.483–23.790)	0.023	
Lung disease, yes vs. none	2.538 (1.091–5.917)	0.031	-	-	
Oliguria (first day), none vs. yes	3.969 (2.002–7.791)	<0.001	-	-	
Diuretic use, none vs. yes	2.034 (1.066–3.883)	0.031	-	-	
RRT used [n (%)], none vs. yes	6.282 (2.205–17.898)	0.001	-	-	
Vasopressor requirement none, vs. yes	143.364 (38.283–536.871)	<0.001	21.067 (3.287–135.009)	0.001	
Sepsis, none vs. yes	5.458 (2.668–11.169)	<0.001	-	-	
Mechanical ventilation, none vs. yes	75.614 (9.072–630.221)	<0.001	37.672 (25.595–399.298)	<0.001	

## Data Availability

The data that support the findings of this study are available from the corresponding author upon reasonable request.

## Abbreviations

The following abbreviations are used in this manuscript:

AKI	Acute kidney injury
ICU	Intensive care unit
GCS	Glasgow Coma Scale
APACHE II	Acute Physiology and Chronic Health Evaluation
RRT	Renal replacement therapy
MV	Mechanical ventilation
LOS	Length of stay
BUN	Blood urea nitrogen
LDH	Lactic dehydrogenase
Crp	C-reactive protein
APTT	Activated partial thromboplastin time
INR	International normalized ratio
BAR	BUN/albumin

## References

[1] Kellum J.A., Romagnani P., Ashuntantang G., Ronco C., Zarbock A., Anders H.J. (2021). Acute kidney injury. Nat. Rev. Dis. Primers.

[2] Silver S.A., Long J., Zheng Y., Chertow G.M. (2017). Cost of acute kidneyinjury in hospitalized patients. J. Hosp. Med..

[3] Becker S., Muller J., de Heer G., Braune S., Fuhrmann V., Kluge S. (2015). Clinical characteristics and outcome of very elderly patients ≥90 years in intensive care: A retrospective observational study. Ann. Intensive Care.

[4] Kane-Gill S.L., Sileanu F.E., Murugan R., Trietley G.S., Handler S.M., Kellum J.A. (2015). Risk factors for acute kidney injury in older adults with critical illness: A retrospective cohort study. Am. J. Kidney Dis..

[5] Liu B., Lv D. (2023). Prognostic value of C-reactive protein to albumin ratio for mortality in acute kidney injury. BMC Nephrol..

[6] Deng Y., Li X., Lai Q., Wang F., Zhang C., Yang Y., Jiang D., Kang H., Wang H., Liao D. (2023). Prognostic implication of lactic dehydrogenase-to-albumin ratio in critically ill patients with acute kidney injury. Clin. Exp. Nephrol..

[7] Haines R.W., Zolfaghari P., Wan Y., Pearse R.M., Puthucheary Z., Prowle J.R. (2019). Elevated urea-to-creatinine ratio provides a biochemical signature of muscle catabolism and persistent critical illness after major trauma. Intensive Care Med..

[8] Sullivan D.H., Sullivan S.C., Bopp M.M., Roberson P.K., Lensing S.Y. (2018). BUN as an Independent Predictor of Post-Hospital-Discharge Mortality among Older Veterans. J. Nutr. Health Aging.

[9] Belinskaia D.A., Voronina P.A., Shmurak V.I., Jenkins R.O., Goncharov N.V. (2021). Serum Albumin in Health and Disease: Esterase, Antioxidant, Transporting and Signaling Properties. Int. J. Mol. Sci..

[10] Robinson R. (2015). Low serum albumin and total lymphocyte count as predictors of 30 day hospital readmission in patients 65 years of age or older. PeerJ.

[11] Yang F., Wang R., Lu W., Hu H., Li Z., Shui H. (2023). Prognostic value of blood urea nitrogen to serum albumin ratio for acute kidney injury and in-hospital mortality in intensive care unit patients with intracerebral haemorrhage: A retrospective cohort study using the MIMIC-IV database. BMJ Open.

[12] Shi Y., Duan H., Liu J., Shi X., Zhang Y., Zhang Q., Zhao M., Zhang Y. (2024). Blood urea nitrogen to serum albumin ratio is associated with all-cause mortality in patients with AKI: A cohort study. Front. Nutr..

[13] Bae S.J., Kim K., Yun S.J., Lee S.H. (2021). Predictive performance of blood urea nitrogen to serum albumin ratio in elderly patients with gastrointestinal bleeding. Am. J. Emerg. Med..

[14] Acehan S. (2024). Acute kidney injury and COVID-19: The predictive power of BUN/albumin ratio for renal replacement therapy requirement. Ir. J. Med. Sci..

[15] Duarte I., Gameiro J., Resina C., Outerelo C. (2020). In-hospital mortality in elderly patients with acute kidney injury requiring dialysis: A cohort analysis. Int. Urol. Nephrol..

[16] Akposso K., Hertig A., Couprie R., Flahaut A., Alberti C., Karras G.A., Haymann J.P., Costa De Beauregard M.A., Lahlou A., Rondeau E. (2000). Acute renal failure in patients over 80 years old: 25-years’ experience. Intensive Care Med..

[17] Kohli H.S., Bhat A., Aravindan A.N., Sud K., Jha V., Gupta K.L., Sakhuja V. (2007). Predictors of mortality in elderly patients with acute renal failure in a developing country. Int. Urol. Nephrol..

[18] Shi X., Zhong L., Lu J., Hu B., Shen Q., Gao P. (2024). Clinical significance of the lactate-to-albumin ratio on prognosis in critically ill patients with acute kidney injury. Ren. Fail..

[19] Gong Y., Zhang F., Ding F., Gu Y. (2012). Elderly patients with acute kidney injury (AKI): Clinical features and risk factors for mortality. Arch. Gerontol. Geriatr..

[20] Liu J., Min J., Lu J., Zhong L., Luo H. (2024). Association between lactate/albumin ratio and prognosis in critically ill patients with acute kidney injury undergoing continuous renal replacement therapy. Ren. Fail..

[21] Kazory A. (2010). Emergence of blood urea nitrogen as a biomarker of neurohormonal activation in heart failure. Am. J. Cardiol..

[22] Chen C.Y., Lin Y.R., Zhao L.L., Yang W.C., Chang Y.J., Wu H.P. (2013). Clinical factors in predicting acute renal failure caused by rhabdomyolysis in the ED. Am. J. Emerg. Med..

[23] Roche M., Rondeau P., Singh N.R., Tarnus E., Bourdon E. (2008). The antioxidant properties of serum albumin. FEBS Lett..

[24] Don B.R., Kaysen G. (2004). Serum albumin: Relationship to inflammation and nutrition. Semin. Dial..

[25] Artero A., Zaragoza R., Camarena J.J., Sancho S., González R., Nogueira J.M. (2010). Prognostic factors of mortality in patients with community-acquired bloodstream infection with severe sepsis and septic shock. J. Crit. Care.

[26] Vincent J.L., Dubois M.J., Navickis R.J., Wilkes M.M. (2003). Hypoalbuminemia in acute illness: Is there a rationale for intervention? A meta-analysis of cohort studies and controlled trials. Ann. Surg..

[27] Contreras A.M., Ramírez M., Cueva L., Alvarez S., de Loza R., Gamba G. (1994). Low serum albumin and the increased risk of amikacin nephrotoxicity. Rev. Invest. Clin..

[28] Murashima M., Nishimoto M., Kokubu M., Hamano T., Matsui M., Eriguchi M., Samejima K.I., Akai Y., Tsuruya K. (2019). Inflammation as a predictor of acute kidney injury and mediator of higher mortality after acute kidney injury in non-cardiac surgery. Sci. Rep..

[29] Wiedermann C.J., Wiedermann W., Joannidis M. (2010). Hypoalbuminemia and acute kidney injury: A meta-analysis of observational clinical studies. Intensive Care Med..

[30] Han T., Cheng T., Liao Y., Tang S., Liu B., He Y., Gu Z., Lei C., Cao Y., Cao Y. (2022). Analysis of the Value of the Blood Urea Nitrogen to Albumin Ratio as a Predictor of Mortality in Patients with Sepsis. J. Inflamm. Res..

[31] Dundar Z.D., Kucukceran K., Ayranci M.K. (2021). Blood urea nitrogen to albumin ratio is a predictor of in-hospital mortality in older emergency department patients. Am. J. Emerg. Med..

[32] Pan Q., Peng Y., Ni H., Lin L., Luo B., Huang X., Chen L., Lin Y. (2024). Blood-urea-nitrogen-to-serum-albumin ratio in predicting the value of patients with contrast-induced nephropathy for coronary heart disease. Int. Urol. Nephrol..

[33] Ye L., Shi H., Wang X., Duan Q., Ge P., Shao Y. (2022). Elevated Blood Urea Nitrogen to Serum Albumin Ratio Is an Adverse Prognostic Predictor for Patients Undergoing Cardiac Surgery. Front. Cardiovasc. Med..

[34] Xiao Q., Zhou Q., Shen W., Dong S., Tan Y., Zhang X., Yao L., Li Q., Qin Z., Wang T. (2024). Blood urea nitrogen-to-albumin ratio independently predicts 30-day mortality in acute respiratory failure patients: A retrospective cohort study. J. Thorac. Dis..

[35] Li J., Peng J., Cheng C., Zhang J., Li L. (2025). Association Between Blood Urea Nitrogen to Serum Albumin Ratio and Mortality in Critically Ill Patients With Chronic Obstructive Pulmonary Disease: A Retrospective Study. Int. J. Chron. Obstruct Pulmon Dis..

[36] He T., Li G., Xu S., Guo L., Tang B. (2022). Blood Urea Nitrogen to Serum Albumin Ratio in the Prediction of Acute Kidney Injury of Patients with Rib Fracture in Intensive Care Unit. Int. J. Gen. Med..

